# Nutritional and sensory characteristics of developed wheat burger buns incorporated with different levels of steamed-squid (*Loligo forbesii*) powder

**DOI:** 10.3389/fnut.2025.1615629

**Published:** 2025-05-22

**Authors:** Rehab F. M. Ali, Ayman M. El-Anany

**Affiliations:** ^1^Department of Food Science and Human Nutrition, College of Agriculture and Food, Qassim University, Buraydah, Saudi Arabia; ^2^Special Food and Nutrition Department, Food Technology Research Institute, Agricultural Research Center, Giza, Egypt

**Keywords:** burger buns, squid (*Loligo forbesii*), diet, nutritional characteristics, techno-functional characteristics, sensory characteristics

## Abstract

**Introduction:**

The main aim of the present investigation was to determine the nutritional and sensory characteristics of wheat flour burger buns supplemented with different quantities of steamed squid powder (SSP).

**Methods:**

Wheat flour was partially substituted with SSP at various proportions (0, 1, 2, 3, 4, and 5% w/w). The proximate composition and techno-functional features of wheat flour, SSP, and composite flours were investigated. Chemical composition, specific volume, amino acid profile, and sensory characteristics of developed burger buns were investigated to determine the most suitable levels of substitution.

**Results:**

The techno-functional characteristics of produced composite flours were significantly improved by inclusion different levels of SSP into wheat flour. The incorporation of SSP into burger buns significantly enhances protein content while reducing carbohydrate levels. The study indicates that as the percentage of SSP increases from 1 to 5%, the protein content of the buns increases correspondingly, with a maximum increase of 1.42 times at 5% SSP. Additionally, carbohydrate levels decrease from 82.06% in control samples to 76.16% with the highest SSP addition. However, the specific volume of the buns shows a decline at higher SSP concentrations, particularly at 4 and 5%, where volumes drop to 2.89 and 2.85 cm^3^ g^−1^, respectively. The incorporation of SSP into burger buns significantly enhances the nutritional profile, particularly in essential amino acids. While the total essential amino acids and specific amino acids like leucine, isoleucine, and valine exceed FAO/WHO requirements, lysine levels remain a concern, increasing only to 3.20 g per 100 g of protein, below the required 5.2 g. The sensory evaluation of these buns shows high acceptability, with scores between 8.38 and 8.61, indicating a positive consumer response. Originality/value—The incorporation of steamed squid powder (SSP) into burger buns has been shown to enhance both nutritional and sensory properties, particularly when substituting 1–3% of wheat flour with SSP. This approach aligns with trends in food innovation aimed at improving health benefits while maintaining consumer acceptance.

## Introduction

The incorporation of protein-rich ingredients into burger buns is a growing trend aimed at enhancing their nutritional profile. This shift addresses dietary deficiencies and promotes better health outcomes by increasing protein content and improving the overall quality of staple foods. Various studies highlight the benefits of using different protein sources, including plant-based options, to fortify burger buns products effectively (1, 2). Incorporating ingredients like soybean flour, barley flour, and texturized vegetable proteins (TVP) significantly boosts the protein content of wheat buns. For instance, replacing 35% of wheat flour with TVP can increase protein content by 83% (3). Higher protein intake from enriched buns can improve body resistance and immunity, making them beneficial for various populations, including older adults (4). The addition of protein-rich ingredients can affect dough properties and final burger buns characteristics (5). The use of milk and whey protein concentrates has shown promise, with studies indicating that burger buns containing 9% milk protein concentrate can increase protein content by 35% compared to control samples (6). However, whey protein may negatively affect dough rheology (6). Legumes and pulses are effective for enriching burger buns, providing a balanced amino acid profile and additional nutrients (7). For instance, watermelon seed flour has been successfully integrated into low-carbohydrate, high-protein burger buns, enhancing its nutritional value (8). Ingredients like poppy seed flour can also be used to create low-carbohydrate, high-protein burger buns, yielding a product with significant protein content (16.3%) and fiber (10.2%) (9). The enrichment of burger buns with protein sources not only addresses protein deficiencies but also supports metabolic health, particularly for individuals with impaired glucose metabolism (10). This approach aligns with dietary recommendations for balanced nutrition.

In this regard, Cephalopods, particularly squid, play a crucial role in marine ecosystems (11), serving as both predators and prey while also being significant for human consumption (12, 13). Their increasing importance is reflected in the rising global fish consumption, which highlights the demand for marine products as a source of animal protein (14). Cephalopods are recognized for their high protein content, which is essential for growth and energy demands (15). Studies indicate that these marine organisms efficiently absorb and utilize dietary proteins, which are crucial for their metabolic processes (16). The amino acid profiles of these cephalopods demonstrate a balanced ratio of essential to non-essential amino acids, enhancing their biological value (17).

Squids are a vital resource in global fisheries, with an estimated catch of 2.1 million tons per year, reflecting their high productivity and growth rates (18). Their nutritional benefits, including high protein and low-fat content, make them a healthy dietary choice (19). However, the intrinsic hard structure of squid meat poses challenges in culinary applications (20). The hard structure of squid meat can complicate cooking and preparation, necessitating specific techniques to ensure tenderness (19). The texture of squid meat is significantly influenced by its collagen content, particularly myostromin, which can make it tough and challenging to chew (21). Various processing methods have been explored to enhance tenderness (20). Cooking squid for over 1 min at 100°C gelatinizes collagen, improving tenderness, but exceeding 5 min can lead to hardening (22). While ultrasound waves can achieve desired tenderization, consumer safety concerns limit its acceptance (23). The composition of squid powder, as reported by Loppies et al. (24), reveals its significant nutritional value, particularly in protein content. Squid powder contains 72.28%% protein, making it a rich source of essential amino acids, which are crucial for various bodily functions. Therefore, the main objective of the present investigation was to assess the nutritional and sensory attributes of wheat flour burger buns incorporated with different levels of steamed-quid (*Loligo forbesii*) powder.

## Materials and methods

### Raw material and chemicals

Fresh squid (*Loligo forbesii*) samples were bought at Nesto Hypermarket, buraydah, Qassim in March 2025. Superior Wheat flour (70% extraction) was obtained from First Mills company, Saudi Arabia. Bakemate instant dry yeast (*Saccharomyces cerevisiae*), Express bread improver (composition: wheat bread flour, enzymes (microbial alpha-amylase), ascorbic acid, calcium carbonate, diacetyl tartaric acid, esters of mono- and di-glycerides of fatty acids), (Egypt), palmolein oil, sugar, salt, were obtained from a local market in Buraydah, Qassim, Saudi Arabia. All of the chemicals used in the present study were of analytical grade.

### Preparation of steamed-squid powder (SSP)

Fresh squid samples were cleaned to eliminate guts, ink, and other impurities. After weeding, the fresh squid was rinsed with running water. The samples were sliced. 200 g of squid slices placed a stainless-steel steamer with a lid, above 1,000 mL of boiling water for 5 min. Steamed slices were drained on a stainless sieve until cold and dried in an electric oven at 70°C for 72 h. The dried materials were pulverized using an electric grinder (Braun Model 1021) and sieved through a 0.7 mm mesh.

### Preparation of wheat flour SSP composite flours

Wheat flour was partially substituted with SSP at different levels (0, 1, 2, 3, 4, and 5% w/w). To ensure homogeneity, the composite flours were dry mixed in a BLACK+DECKER Kitchen Stand dough mixer (Model 2724607829595) for 15 min. The resulting mixture was sealed in polypropylene bags and refrigerated at (4 ± 1°C) for subsequent use. These combinations (composite flours) were developed through initial assessment experiments (unpublished data).

### Preparation of wheat burger buns

Table 1 shows the ingredients of traditional and developed doughs. The straight dough method was applied in compliance with the procedures described by Ali et al. (1). Water addition was based on farinogram water absorption (500 FU) (Table 1). The flour, Improver, dry yeast, powdered sugar, salt, and half of the oil were then added, followed by 5 min of mixing at slow speed. Immediately after that, half the remaining amount of oil was added and the dough mixer was operated on high speed for 4 min. The dough was allowed to rest for 5 min. After resting time, the rested dough was shaped into 80 g balls. The dough balls were and placed in baking pans and proofed at 38°C and 85% RH for 60 min. The baking procedure was carried out using a Real Forni oven (BE 4C12.30, Italy) at 250°C for 11 min. Burger buns were cooled in a cooling chamber at 25 C for 2 h before being packaged into polyethylene bags. After 3 h of cooling process, the wheat burger buns were evaluated for sensory quality and specific volume.

**Table 1:** Formulation of wheat burger buns dough incorporating different quantities of squid powder (SP).

Ingredients	% Wheat flour (% additives)	Superior Wheat flour 100%	SWF: SSP (w/w)				
			99:1	98:2	97:3	96:4	95:5
Superior Wheat flour (g)	100	1,000	990	980	970	960	950
Steamed squid powder (SSP) (g)	–	–	10	20	30	40	50
Water (mL)	60	600	611	620	628.5	635	643
Dry active yeast (g)	1.25	12.5	12.5	12.5	12.5	12.5	12.5
Sugar (g)	6	60	60	60	60	60	60
Salt (g)	1	10	10	10	10	10	10
Improver (g)	0.50	5.0	5.0	5.0	5.0	5.0	5.0
Palmolein oil (mL)	3.00	30	30	30	30	30	30

Superior Wheat flour (SWF), Steamed squid powder (SSP).

### Proximate composition analysis

Proximate analysis was performed in accordance with AOAC standards (87). Moisture content was assessed using the air draft oven technique (AOAC method number 925.09B). The crude protein was determined using the Micro-Kjeldahl method following AOAC method no. 950.36. The ether extract (fat) content was measured using the Soxhlet extraction technique (AOAC method no. 950.36). The crude fiber content was determined using a digestion technique (AOAC method no. 950.37), as well as the ash content was assessed using the dry ashing approach (AOAC method no. 930.22). The carbohydrate content was calculated by subtracting the total of proximal components from 100% (1).

Energy value (kcal/100 g) was calculated as shown in the following equation (2).


$$\begin{array}{l} \text{Energy}\;(\text{kcal}/100\;g)=[(9\times \text{lipids}\%)+(4\times \text{proteins}\%)\\ +(2\times \text{fiber}\%)+(4\times \text{carbohydrates}\%)] \end{array}$$

### Specific volume of loaf

The ratio of the loaf volume (LV) to its weight was used to determine the specific volume (SV) of the loaves. A sensitive electronic digital weighing balance was used to determine the loaf’s weight. The rapeseed displacement method was used to measure the LV (25).

### Techno-functional properties of wheat flour, squid powder (SP) and composite flours

The water absorption capacity (WAC %) of original and composite flours was determined using the Sosulski et al. (26) technique. One gram of flour sample was mixed with 10 mL distilled water and allow to remain at ambient temperature (30 ± 2°C) for 30 min, the mixture was centrifuged for 30 min at 3,000 rpm or 2,000×*g*. Water absorption was measured as percent water bound per gram flour. The oil absorption capacity (OAC%) was also determined using the Sosulski et al. (26) technique. After mixing 1 g of sample with 10 mL of soybean oil (Sp. Gravity: 0.9092), it was allowed to remain at ambient temperature (30 ± 2°C) for 30 min before being centrifuged for 30 min at 300 rpm or 2,000×*g*. Oil absorption was measured as a percentage of oil bound per gram flour. The emulsion activity (EA, %) was measured according to procedures described by Yasumatsu et al. (88). In the current investigation, an emulsion (1 g sample, 10 mL distilled water, and 10 mL soybean oil) was prepared in a calibrated centrifuge tube. The emulsion was centrifuged at 2,000×*g* for 5 min. The ratio of the height of the emulsion layer to the total height of the mixture was calculated to determine emulsion activity in percentage. The foam capacity (FC, %) was measured using the techniques given by Narayana and NarsingaRao (27). One gram of flour was mixed with 50 mL of distilled water at 30 ± 2°C in a graduated cylinder. The suspension was combined and shook for 5 min to produce foam. The volume of foam 30 s after whipping was represented as foam capacity using the following equation:


$$\begin{array}{l} \text{Foam capacity}\;(\%)=[\text{volume of foam}\;\mathrm{AW}\\ -\text{Volume of foam}\;\mathrm{BW}]/\text{volume of foam}\;\mathrm{BW}\times 100. \end{array}$$

Where, AW = after whipping, BW = before whipping.

### Amino acids analysis

High-performance liquid chromatography (HPLC) was used to determine amino acid levels, following the method mentioned by Alajaji and El-Adawy (28). Hydrolysis was performed using 6 M HCl at 110°C for 24 h, under an atmosphere of nitrogen. Performic acid oxidation was used to identify sulfur-containing amino acids. The analysis of tryptophan content was conducted in accordance with the procedure described by Miller (29). Amino Acid Score (AAS) was estimated using sample amino acid compositions as [mg of amino acid in 1 g of test protein/mg of amino acid in requirement pattern] × 100 (30). To determine the overall score, the lowest AAS determined represents the protein source’s first-limiting amino acid (30).

### Sensory evaluation

The sensory evaluation of burger buns, was performed as authorized by the Qassim University Ethics Committee. Before the study commenced, panelists provided informed, written consent and were invited to a sensory evaluation training session for Check-All-That-Apply (CATA) products. Informed consent ensures ethical standards are met, allowing participants to understand the study’s purpose and their role. Training sessions equipped panelists with the necessary skills to identify and articulate sensory attributes effectively. A panel of 45 untrained burger buns consumers (19–55 years old, 30 females and 15 males) were assessed for acceptability using a 9-point hedonic scale 1 (extreme detest) to 9 (extreme preference) (31). Consumers were recruited from different departments of Qassim University. Burger buns samples (10 g) were placed on pre-prepared and coded disposable paper trays. To offset flavor differences between samples, pure water bottles at 20°C were employed. The evaluation of burger buns was conducted in a temperature-controlled environment at 25°C, utilizing daylight fluorescent lights. Different attributes appearance, crumb color, odor, taste, texture by mouth feel, and overall acceptance were evaluated.

### Statistical analysis

SPSS software version 21.0 (Chicago, IL, USA) was implemented to perform statistical analyses of the data (five replicates, *n* = 5), with the exception of sensory results, *n* = 45. In accordance with the methodology outlined by Gomez and Gomez (32), data were examined using Duncan’s new multiple range tests to identify the significant differences subsequent to one-way analysis of variance (ANOVA) at *p* < 0.05.

## Results and discussion

### Proximate composition of superior wheat flour (SWF) and steamed squid powder (SSP) (g per 100 g dry weight basis)

The chemical composition of superior wheat flour (SWF) and SSP reveals significant differences (Table 2). The moisture content of SSP is significantly low (8.50%), which enhances its storage capabilities and extends its shelf life, making it a safer option for food powders (1). The high protein content of SSP (78.98%) aligns with findings from various studies that highlighted the nutritional value of squid-derived products. A study indicated that squid flour (*Loligo* sp.) contains approximately 73.87% protein, which is comparable to the reported levels in squid powder (33). Another study focused on protein concentrates derived from jumbo squid viscera, which also demonstrated substantial protein levels, further supporting the high protein claims of squid products (34). SSP is characterized by its moderate fat, ash, and carbohydrate content, measured at 3.90, 3.75, and 13.37%, respectively. This composition makes SSP a valuable ingredient in various food applications. The moderate fat content in SSP contributes to the energy density of diets. Fat powder derived from squid has shown to enhance lipid oxidation stability in fish diets (35). The ash content indicates the mineral presence in SSP (36). SSP enhances the nutritional profile of products like dried noodles, improving their taste and health benefits (36). Table 2 shows also that superior wheat flour is primarily composed of carbohydrates (86.96%) and contains only 10.60% crude protein, therefore the incorporation of SSP into superior wheat flour can significantly enhance the nutritional quality of burger buns.

**Table 2:** Proximate composition of superior wheat flour (SWF) and steamed squid powder (SSP) (g per 100 g dry weight basis).

Components (g/100 g dry weight basis)	Superior Wheat flour (SWF)	Steamed squid powder (SSP)
Moisture	11.50^a^ ± 0.98	8.50^b^ ± 0.77
Crude protein	10.60^b^ ± 0.91	78.98^a^ ± 0.66
Fat content	1.25^b^ ± 0.09	3.90^a^ ± 0.07
Crude fibers	0.59^a^ ± 0.08	ND
Ash	0.60^b^ ± 0.09	3.75^a^ ± 0.07
Total carbohydrates	86.96^a^ ± 1.65	13.37^b^ ± 0.87
Energy value (kCal/100 g)	401.49^a^ ± 0.60	404.50^a^ ± 0.41

Values are means ± SD of five determinations.

Means in the same row with different letters are significantly different (*p* ≤ 0.05).

### Techno-functional properties of wheat flour, steamed squid powder (SSP) and their composite flours

Table 3 shows the result of some techno-functional properties of wheat flour, steamed squid powder (SSP) and their composite flours. Water absorption capacity (WAC) of flour samples ranged from 145.00 to 480.00% (Table 3). The WAC of flour is a critical functional property that influences the quality and texture of food products (37). The WAC of flour samples varied significantly based on their composition, particularly the protein content. The higher WAC of SSP (480.00%) is attributed to its high protein content (78.98%), which enhances its ability to retain water. This is in contrast to wheat flour, which has a lower WAC (145.00%) due to its lower protein content. High protein content, as seen in SSP, significantly enhances WAC. Proteins have hydrophilic properties that allow them to bind water effectively, which is evident in the high WAC of SSP compared to wheat flour (38). The incorporation of SSP into wheat flour significantly enhances water absorption capacity (WAC), as evidenced by the increased WAC values at varying percentages of SSP. The WAC values of wheat flour with 1–5% SSP were found to be 1.02–1.11 times greater than that of the control, indicating a clear positive correlation between SSP concentration and WAC (Table 3). Increased WAC can improve the texture and moisture retention of baked products, potentially leading to better sensory qualities (39). Composite flours often exhibit significantly higher protein content compared to traditional wheat flour. In this respect, blends containing legumes and other protein-rich ingredients have shown protein levels ranging from 15.81 to 21.98% (38). In this regard, composite flours made from aerial yam and wheat demonstrated higher WAC values (113.50–134.00 g/mL) compared to 100% wheat flour (102.50 g/mL) (40). The protein’s hydrophilic nature contributes to this increase, as proteins can bind more water, enhancing the flour’s ability to absorb moisture (41).

**Table 3:** Techno-functional properties of wheat flour, steamed Squid powder (SSP) and composite flours.

Techno-functional properties	Superior Wheat flour (SWF)	Steamed squid powder (SSP)	SWF SSP (w/w)				
			99:1	98:2	97:3	96:4	95:5
WAC (%)	145.00^e^ ± 2.21	480.00^a^ ± 3.10	149.00^de^ ± 1.87	151.90^d^ ± 2.19	156.00^c^ ± 2.66	158.60^bc^ ± 1.78	162.00^b^ ± 1.33
OAC (%)	147.01^e^ ± 2.34	320.00^a^ ± 4.01	150.10^de^ ± 1.77	151.80^d^ ± 1.87	152.30^c^ ± 1.14	154.10^b^ ± 1.70	155.75^b^ ± 1.65
EA (%)	35.13^f^ ± 1.78	178.00^a^ ± 2.22	36.90^e^ ± 1.12	38.60^d^ ± 1.98	39.80^c^ ± 1.45	41.00^b^ ± 1.68	42.70^b^ ± 0.98
FC (%)	14.05^g^ ± 0.44	249.00^a^ ± 2.00	16.50f ± 3.41	18.90^e^ ± 2.60	21.25^d^ ± 1.39	24.10^c^ ± 1.56	26.80^b^ ± 0.99

Values are means ± SD of five determinations.

Means in the same row with different letters are significantly different (*p* ≤ 0.05).

Water absorption capacity (WAC), oil absorption capacity (OAC), emulsion activity (EA), and foam capacity (FC).

The oil absorption capacity of SSP is significantly higher than that of super wheat flour, attributed to the distinct chemical composition of these materials. The high oil absorption of SSP, at 320.00%, can be linked to its rich protein and lipid content, which enhances its ability to bind oils compared to the lower absorption of wheat flour at 147.00% (Table 3). SSP contains a high percentage of protein, which contributes to its oil-binding capacity (42). The method of preparing SSP, involving steaming and drying, optimizes its nutritional profile and oil absorption properties (36). The binding of proteins to lipids can enhance the functional characteristics of food products, including texture and flavor (43). Hydrophobic amino acids form interactions with lipids, which can improve the emulsifying properties of proteins in food systems (44). The heat denaturation of proteins in SSP may enhance their emulsifying properties, which is crucial for maintaining the quality of bakeries (44). The unique protein-lipid interactions in SSP can contribute to improved moisture retention and shelf-life of bakeries (43).

The OAC of superior wheat flour (SWF) increased from 147.00 to 155.75% with the addition of 5% SSP, indicating a positive correlation between SSP levels and OAC. Higher protein content in composite flours contributes to improved OAC, as proteins can interact with lipids, enhancing emulsification and moisture retention (45). Similar studies show that blending wheat flour with other flours, such as fenugreek, and *Chlorella vulgaris* powder also increases OAC and enhances sensory attributes, demonstrating the versatility of composite flours (1, 46).

The emulsion activity (EA%) of flour samples, ranging from 35.13 to 178.00%, highlights significant variations in emulsifying properties among different flour types. The highest EA% was observed in SSP, while Superior Wheat flour (SWF) exhibited the lowest (Table 3). SSP contains proteins and lipids that enhance emulsification, leading to a higher EA% due to better interfacial interactions and stability of emulsions (47). Lower protein content and gluten quality may result in reduced emulsifying capacity, as indicated by its lower EA% (48). The presence of specific proteins and carbohydrates in flours affects their emulsifying properties. For instance, emulsifiers derived from flour can improve stability against physical and chemical deterioration (49). The method of flour processing, such as milling and heating, can alter the functional properties of emulsifiers, impacting EA% (50). The incorporation of SSP into composite flours significantly enhances emulsion activity (EA%), as evidenced by an increase from 35.13 to 42.70% with just 5% SSP addition. This improvement is attributed to the small molecular weight proteins present in the squid powder, which facilitate effective emulsion formation through rapid diffusion to interfaces (36). Composite flours, including various powders like migratory locust (*Locusta migratoria*) powder exhibit improved functional properties such as swelling capacity and water absorption (2). The use of composite flours in products like biscuits and noodles shows enhanced sensory qualities and nutritional benefits (51, 52).

The foam capacity values indicate significant variability among different flour samples, with SSP exhibiting the highest foam capacity at 249%, while refined wheat flour showed the lowest at 14.05% (Table 3). SSP demonstrated exceptional foam capacity (249%), attributed to its protein composition and structural properties, which enhance air incorporation and stability (34). SWF exhibited a low foam capacity (14.05%), indicating limited ability to stabilize foams compared to protein-rich alternatives (53). The use of ultrasonicated squid ovary powder (USOP) as a substitute for egg white powder (EWP) in cake formulations has shown promising results in enhancing batter and cake properties. Research indicates that substituting EWP with USOP at varying levels (12.5–100%) significantly affects the textural, structural, and sensory attributes of the final product (54). The proteins from squid viscera have shown good emulsifying and foaming properties, which can be exploited in various food applications (34). Higher overall liking scores were reported for cakes with 100% USOP, particularly in terms of firmness (54). The incorporation of SSP significantly enhances foaming capacity (FC %) in composite flours, as evidenced by increases of 7.20, 10.05, and 12.75% with 3, 4, and 5% SSP additions, respectively. This improvement is attributed to various factors influencing foam formation, including protein hydrophobicity, amino acid distribution, and the presence of activated sulfhydryl groups. Proteins with higher hydrophobic regions tend to stabilize foams better, as they reduce surface tension and promote bubble formation (55). The arrangement of non-polar amino acids on protein surfaces plays a crucial role in foam stability, affecting how proteins interact with air and water (56). Activated sulfhydryl groups can enhance protein interactions, leading to stronger foam structures (57). The amphiphilic nature of proteins, is vital for creating stable foams in baked goods, contributing to texture and volume (2). The use of vegetable proteins and other biological particles is gaining traction for their foaming properties, offering sustainable options for food formulations (58).

These findings concluded that the incorporation of SSP into wheat flour significantly enhances various functional properties, including water absorption capacity (WAC), oil absorption capacity (OAC), emulsion activity (EA%), and foaming capacity (FC%). These improvements contribute to better texture, moisture retention, and overall quality of baked products.

### Chemical composition and physical characteristics of burger buns incorporated with different quantities of steamed squid powder (SSP)

The proximate composition and physical properties of developed burger buns fortified with SSP are significantly influenced by the substitution ratio of the squid powder (Table 4). The moisture content of burger buns is significantly influenced by the addition of SSP. The study indicates that as the percentage of SSP increases, the moisture content of the burger buns also rises, with the highest recorded at 40.45% for samples containing 5% SSP. The increased moisture content attributed to SSP proteins compared to wheat flour can be explained through several key factors. The superior water absorption capacity of SSP is primarily due to its unique protein composition and structure, which enhances its functional properties in food applications (Tables 2, 3). The proteins derived from squid proteins, which have been shown to possess good emulsifying and foaming properties, enhancing moisture retention in food products (34). The steaming and drying process of squid powder increases the availability of free amino acids and unsaturated fatty acids, which can further improve water absorption during cooking (36). Higher moisture levels can enhance the texture and freshness of burger buns, potentially improving consumer acceptance (59). The addition of SSP to burger buns formulations significantly increases the protein content, as demonstrated by the range of 12.13–17.31% protein content in burger buns samples with varying levels of SSP (Table 4). Burger buns with 1, 2, 3, 4, and 5% SSP showed protein increases of 1.08, 1.18, 1.28, 1.35, and 1.42 times, respectively, compared to the control. This increase is attributed to the high protein content of SSP, which constitutes 78.98% of its chemical composition (Table 2). The enhancement of protein content in burger buns through the incorporation of non-traditional protein sources is a well-documented approach in food science, aiming to improve nutritional profiles and meet dietary needs. The study of bread enriched with fish protein, such as minced fish, shows similar trends where protein content increases with the addition of fish protein. For instance, bread samples with 3 and 5% dry washed minced fish (DWMF) showed protein increases of 31 and 45%, respectively, compared to bread samples without fish protein (60). High-protein formulations often include a variety of protein sources, such as soybeans and beef, to achieve a balanced amino acid profile and enhance nutritional value (61). The incorporation of SSP into burger buns formulations significantly influences the fat content. The addition of SSP at 3, 4, and 5% resulted in fat contents of 4.25, 4.33, and 4.41%, respectively, indicating a direct correlation between SSP concentration and fat content in the burger buns. This enhancement in fat content can be attributed to the nutritional profile of squid, which is rich in lipids and proteins, thereby enriching the burger buns’s overall composition. Squid is known for its high protein and essential fatty acids, which can improve the nutritional value of burger buns, aligning with consumer trends toward healthier options (62). The results showed that there are no significant differences in crude fiber content among burger buns (Table 4). This finding can be attributed to the absence of fiber in SSP. SSP is primarily composed of proteins and other nutrients but does not contain significant fiber (24). Other studies have shown that incorporating ingredients like mushroom powder can increase crude fiber content significantly, indicating that the choice of additives plays a crucial role in nutritional composition (63). In contrast, SSP’s lack of fiber means it does not contribute to variations in fiber content, unlike other potential additives. The ash content of burger buns ranged from 0.90 to 1.31%, with SSP-enriched samples showing significantly higher values (*p* ≤ 0.05) than the control (Table 4). Specifically, the burger buns with 4 and 5% SSP had ash contents of 1.19 and 1.31%, respectively, indicating a direct correlation between SSP enrichment and ash content. The higher ash content (3.75%) of SSP suggests that incorporating such ingredients can enhance the mineral profile of burger buns, which is typically low in essential nutrients (Table 2). Similar studies have shown that adding fish residues to bread not only increases mineral content but also improves sensory acceptance among consumers (60). The carbohydrate content in baked burger buns significantly influenced by the incorporation of SSP. Results indicated that substituting wheat flour with SSP leads to a significant reduction in carbohydrate levels, with values dropping from 82.06% in control samples to as low as 76.16% with 5% SSP addition. This reduction is attributed to the low carbohydrate content of SSP (13.37%) and its high protein content (78.98%) (Table 2). Similar studies show that using high protein powders, such as those derived from migratory locust (*Locusta migratoria*) powder, can also lower carbohydrate content significantly (2). Incorporating dietary fibers and proteins can further enhance the nutritional profile while reducing carbohydrates (1). The results indicate that there are no significant differences in energy values among burger buns samples, ranging from 415.13 to 415.21 kCal/100 g, this finding can be attributed to the substitution of carbohydrates with protein from SSP. This substitution maintains similar energy values because both carbohydrates and proteins share the same caloric conversion factor, which is approximately 4 kCal per gram.

**Table 4:** Chemical composition and physical characteristics of burger buns incorporated with different quantities of steamed Squid powder (SSP).

Components (g/100 g DW)	SWF 100%	SWF: SSP (w/w)				
		99:1	98:2	97:3	96:4	95:5
Moisture	37.75^f^ ± 1.33	38.26^e^ ± 1.53	38.81^d^ ± 1.77	39.26^c^ ± 0.93	39.82^b^ ± 0.97	40.45^a^ ± 1.36
Protein	12.13^f^ ± 0.92	13.21^e^ ± 0.42	14.34^d^ ± 0.55	15.55^c^ ± 0.35	16.38^b^ ± 0.82	17.31^a^ ± 0.66
Fat	4.08^bc^ ± 0.45	4.15^b^ ± 0.44	4.20^ab^ ± 0.34	4.25^ab^ ± 0.24	4.33^a^ ± 0.19	4.41^a^ ± 0.61
Crude fiber	0.83^a^ ± 0.06	0.83^a^ ± 0.09	0.82^a^ ± 0.05	0.82^a^ ± 0.07	0.81^a^ ± 0.06	0.81^a^ ± 0.04
Ash	0.90^e^ ± 0.03	0.97^d^ ± 0.08	1.07^c^ ± 0.10	1.12^bc^ ± 0.14	1.19^b^ ± 0.09	1.31^a^ ± 0.08
Total carbohydrates	82.06^a^ ± 0.34	80.84^b^ ± 0.25	79.57^c^ ± 0.41	78.26^d^ ± 0.32	77.29^e^ ± 0.52	76.16^f^ ± 0.63
Energy value (kCal/100 g)	415.14^a^ ± 0.37	415.21^a^ ± 0.26	415.08^a^ ± 0.28	415.13^a^ ± 0.22	415.27^a^ ± 0.19	415.19^a^ ± 0.42
Volume (cm^3^)	216.8^a^ ± 2.22	216.8^a^ ± 2.70	216.3^a^ ± 2.37	215.4^ab^ ± 2.19	209.5^b^ ± 2.12	207.0^c^ ± 1.45
Loaf weight (g)	71.15^c^ ± 0.55	71.81^b^ ± 0.37	72.11^b^ ± 0.33	72.30 ^ab^± 0.66	72.50^a^ ± 0.52	72.66 ^a^ ± 0.60
Specific volume (cm^3^ g^−1^)	3.04^a^ ± 0.09	3.01^a^ ± 0.08	3.00^a^ ± 0.09	2.98 ^ab^± 0.10	2.89^b^ ± 0.11	2.85^c^ ± 0.13

Values are means ± SD of five determinations.

Means in the same row with different letters are significantly different (*p* # 0.05).

SWF 100%: control (100% wheat flour).

SSP: steamed Squid powder.

### Physical properties of the bun loaves

The incorporation of SSP into burger buns formulations significantly affects loaf volume, with optimal results observed at lower concentrations. Control samples recorded volumes of 216.8 cm^3^, while 1 and 2% SSP samples maintained similar volumes (216.8, 216.3 cm^3^). A significant decrease in volume was noted at 4% SSP, with the lowest volume of 207 cm^3^ at 5% SSP (Table 4). The dilution of gluten by additional proteins from SSP weakens the gluten network, impairing gas retention and leading to reduced loaf volume (1). Gluten is essential for maintaining the structure and texture of bread, as it traps gases during fermentation. Studies indicate that the weakening of the gluten network correlates with decreased burger buns volume and altered texture characteristics (64–66). Conversely, while SSP enhances certain nutritional aspects of burger buns, excessive incorporation can compromise the desired textural qualities and volume, highlighting the need for careful formulation in burger buns production. The weights of the baked burger buns ranged from 71.15 to 72.66 g (Table 4). The incorporation of SSP into burger buns significantly enhances bun weights. Burger buns with 4 and 5% SSP showed significantly the highest bun weights (*p* < 0.05). SSP’s high water retention capacity (480%) is attributed to its structural components, which can absorb and hold substantial amounts of water, thus contributing to increased loaf weight (Table 2). The presence of water-binding agents enhances the moisture retention in the dough (67). The increased water content can also enhance the dough’s viscosity, which is crucial for achieving the desired texture and structure in the final product (68). Other studies on bread fortification, such as with cod fish powder and legume fibers, also show improvements in nutritional and physical properties, emphasizing the importance of ingredient balance in burger buns formulation (69, 70). The specific bun volumes ranged from 2.85 to 3.04 cm^3^ g^−1^, demonstrates a notable trend where lower concentrations of SSP (1–3%) do not significantly affect this parameter, while higher concentrations (4 and 5%) lead to a significant decrease (2.89 and 2.85 cm^3^ g^−1^, respectively). Higher bun specific volumes correlate with slower staling rates. As specific volume increases, the rate and extent of staling decrease linearly (71). The observed decreases in specific volumes of supplemented burger buns can be attributed to reduced gluten content, which significantly impacts dough structure and gas retention during baking. Several studies corroborate these findings, highlighting the relationship between gluten levels and the physical properties of dough. Higher gluten content enhances gas retention capacity, delaying gas leakage and improving dough stability during proofing (72).

The findings regarding the impact of SSP on bun volumes indicate that lower concentrations (1–3%) do not significantly alter this parameter, while higher concentrations (4–5%) result in a notable decrease. This suggests that incorporating SSP up to 3% of wheat flour is advisable to maintain bun quality.

### Amino acid content (g/100 g protein) of burger buns incorporated with various amounts of steamed squid powder (SSP)

Table 5 illustrates the amino acid content (g/100 g protein) of burger buns incorporated with various amounts of steamed squid powder (SSP). The addition of SSP into burger buns improves their amino acid profile, particularly the essential amino acids. The study showed that the incorporation of different levels of SSP resulted in a marked increase in total essential amino acids, sulfur amino acids, and particular amino acids such leucine, isoleucine, tyrosine, threonine, and valine, exceeding FAO/WHO requirements. However, a 5% supplementation with SSP increased lysine level from 2.90 g to 3.20 g per 100 g of protein, getting below of the FAO/WHO requirement of 5.2 g. The nutritional superiority of steamed squid powder (SSP) over wheat flour proteins is primarily due to its rich essential amino acid profile and high protein content. Studies indicate that SSP contains significant protein concentrations, making it a valuable dietary supplement. SSP typically contains around 73.87% protein, which is higher than many plant-based proteins (33). SSP is recognized for its rich content of essential amino acids, which are vital for tissue growth and repair (73). Wheat flour is notably low in lysine, making SSP a superior alternative for those needing a balanced amino acid profile (73). Incorporating SSP into diets can lead to better overall nutrient profiles, particularly for populations with limited access to diverse protein sources (74). As a marine-derived protein, SSP contributes to sustainable dietary practices, aligning with global goals for responsible consumption (75). The analysis of essential amino acid scores in burger buns reveals that lysine is the primary limiting amino acid (LAA), with control samples showing only 55.76% sufficiency compared to recommended intake levels. Even with the addition of steamed squid powder (SSP), lysine levels improved marginally to 60.38 and 61.53% at 4 and 5% inclusion, respectively, indicating persistent challenges in achieving adequate lysine levels in cereal-based products. Lysine is often the first limiting amino acid in cereal-based diets, as noted in various studies (76). The inclusion of SSP, while beneficial, did not eliminate lysine as the first LAA, indicating a persistent deficiency. The percentages achieved (60.38 and 61.53%) suggest that while SSP can alleviate some deficiency, it is insufficient for complete nutritional adequacy. Conversely, while lysine remains a concern, other amino acids may be present in sufficient quantities, suggesting that a balanced approach to protein sourcing could mitigate overall nutritional deficiencies in such products.

**Table 5:** Amino acids composition (g/100 g protein) of burger buns incorporated with different quantities of steamed squid powder (SSP).

Amino acid	SWF 100%	SWF: SSP (w/w)					FAO/WHO/UNU (30)
		99:1	98:2	97:3	96:4	95:5	
Isoleucine	3.95	3.95	3.96	3.97	3.98	3.98	2.8
Leucine	7.55	7.54	7.54	7.53	7.53	7.52	6.6
Lysine	2.90	2.96	3.02	3.08	3.14	3.20	5.2
Cystine	1.69	1.69	1.69	1.68	1.68	1.68	
Methionine	1.49	1.50	1.52	1.53	1.55	1.56	
Total sulfur amino acids	3.18	3.19	3.21	3.22	3.23	3.24	2.6
Tyrosine	3.10	3.10	3.11	3.15	3.17	3.20	
Phenylalanine	5.84	5.82	5.81	5.79	5.77	5.75	
Total aromatic amino acids	8.94	8.92	8.92	8.94	8.94	8.95	6.3
Threonine	3.34	3.38	3.40	3.43	3.45	3.47	3.4
Tryptophan	1.30	1.29	1.29	1.29	1.29	1.29	1.1
Valine	4.90	4.90	4.90	4.90	4.90	4.91	3.5
Total essential amino acid	36.06	36.13	36.24	36.36	36.46	36.59	
Histidine	2.86	2.85	2.83	2.81	2.8	2.79	
Arginine	5.32	5.32	5.32	5.32	5.32	5.32	
Aspartic acid	6.72	6.70	6.63	6.39	6.21	5.84	
Glutamic acid	22.36	22.38	22.41	22.61	22.76	23.04	
Serine	5.19	5.2	5.22	5.23	5.24	5.26	
Proline	12.95	12.88	12.81	12.74	12.67	12.6	
Glycine	4.7	4.69	4.68	4.67	4.66	4.66	
Alanine	3.84	3.85	3.86	3.87	3.88	3.9	
Total non-essential amino acid	63.94	63.87	63.76	63.64	63.54	63.41	
First limiting amino acid	Lysine (55.76)	Lysine (56.9)	Lysine (58.0)	Lysine (59.2)	Lysine (60.38)	Lysine (61.53)	

SWF 100%: control (100% wheat flour).

SSP, steamed squid powder.

### Sensory attributes of burger buns incorporated with different quantities of steamed squid powder (SSP)

**Figure 1:**
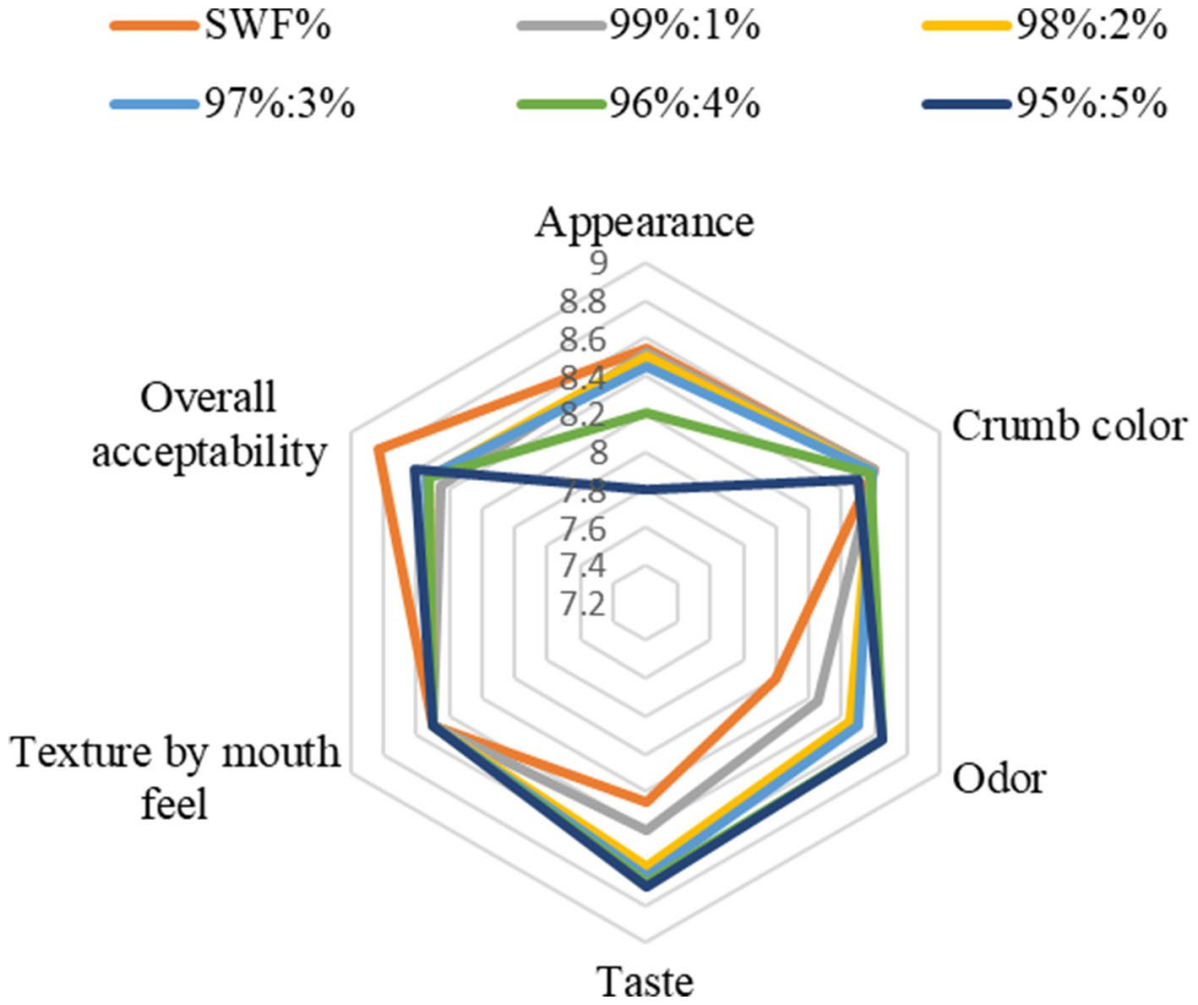
Sensory attributes of burger buns incorporated with different quantities of steamed squid powder (SSP).

Figure 1 shows the sensory attributes of burger buns incorporated with different quantities of steamed squid powder (SSP). The sensory attributes of burger buns, particularly appearance, significantly influence consumer preferences, as evidenced by the varying scores associated with different levels of steamed squid powder (SSP) incorporation. The highest appearance scores were recorded for control samples and those with 1–3% SSP, while higher concentrations (4–5% SSP) resulted in lower scores. Control and 1–3% SSP buns scored between 8.45 and 8.55, indicating strong consumer appeal. In contrast, 4–5% SSP buns scored lower (7.80–8.20), suggesting a threshold beyond which sensory quality declines. This trend aligns with findings from other studies that emphasize the importance of sensory characteristics in food products. Incorporating health-promoting ingredients such as *Chlorella vulgaris* powder, locust powder, salmon fish powder, white shrimp protein hydrolysates and various insect powders into bread can significantly enhance its nutritional profile. However, these additions may adversely affect sensory attributes, particularly appearance, which can influence consumer preferences (1, 2, 77–79). The incorporation of SSP into burger buns has been shown to influence sensory attributes, particularly crumb color and consumer acceptance. While lower concentrations (1–4% SSP) did not significantly alter crumb color compared to control samples, a higher concentration (5% SSP) resulted in darker crust and crumb, leading to lower liking scores among consumers (Figure 1). This phenomenon highlights the delicate balance between nutritional enhancement and sensory quality in food products. The incorporation of SSP in burger buns significantly enhances their odor scores, which is crucial for consumer acceptance. The highest scores recorded (8.65, 8.65, and 8.50 for 5, 4, and 3% SSP, respectively) suggest that the aroma of SSP positively influences sensory perception, leading to higher acceptance rates (Figure 1). Odor is a key sensory attribute that affects food quality and consumer acceptance. Higher odor scores indicate a more favorable sensory experience, which is essential for food products (80). Positive aromas can enhance consumer satisfaction and drive purchase intent. The pleasant scent of SSP likely contributes to a more appealing product, aligning with findings that aroma significantly influences appetite and consumer behavior (81). The positive reception of SSP in burger buns exemplifies how scent can be leveraged to improve product appeal (Figure 1).

Taste is a primary determinant of food acceptability, often outweighing health benefits in consumer choices (82). The incorporation of SSP into burger buns significantly enhances their taste, leading to higher overall acceptability compared to control buns without SSP. Taste scores for buns with varying percentages of SSP (3, 4, and 5%) ranged from 8.65 to 8.70, while the control scored only 8.25 (Figure 1). This improvement can be attributed to the sensory properties of SSP, which align well with consumer preferences for taste in food products. Research indicates that consumers prioritize taste over health benefits, suggesting that products like SSP-enriched buns can meet both sensory and nutritional expectations (83). Texture significantly influences overall food acceptability (84). The observation of no significant differences in mouthfeel among produced burger buns suggests a favorable level of acceptability for these products. This consistency in texture is crucial as it aligns with consumer preferences, which often prioritize mouthfeel as a key attribute in food acceptability. The overall acceptability of burger buns incorporating steamed squid powder (SSP) demonstrates a significant enhancement in sensory properties, with scores ranging from 8.38 to 8.61. The highest scores were attributed to buns with 5, 4, and 3% SSP, indicating a positive consumer response. This suggests that sensory attributes, such as taste and texture, are crucial for product acceptance, aligning with findings that emphasize the importance of sensory characteristics in food evaluation (85, 86). The incorporation of SSP likely improved the flavor profile and texture, leading to higher acceptability scores. The study highlights that consumer liking is influenced by sensory properties, which are critical for market success (85).

### Heat-map of a correlation matrix

**Figure 2:**
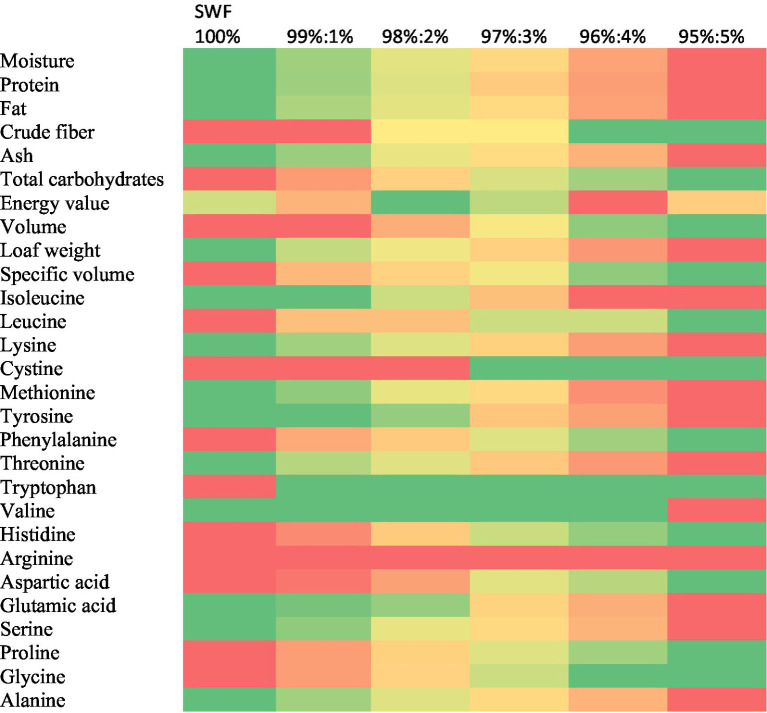
Heat-map of a correlation matrix. Green low, yellow medium, and red high.

The heat-map of a correlation matrix (Figure 2) summarizes the results of the current investigation. This heat map concluded that the incorporation of steamed squid powder (SSP) into burger buns significantly alters their nutritional profile. As the level of SSP increases, there is a notable rise in protein, ash, and fat content, while total carbohydrates decrease. Additionally, the specific volume of the buns diminishes with higher SSP substitution, particularly at 5% levels. The amino acids, including isoleucine, lysine, methionine, tyrosine, threonine, valine, glutamic acid, and alanine, also show significant increases with greater SSP incorporation.

## Conclusion

Steamed squid powder (SSP) is a versatile ingredient with a high protein content of approximately 78.98%, which significantly enhances its functional properties, such as water and oil binding, and emulsifying capabilities. These properties are crucial in improving the quality of bakery products. The incorporation of SSP into wheat flour significantly alters the nutritional profile and baking characteristics of bread. At substitution levels of 1–3%, SSP enhances protein, mineral, and fat contents while reducing carbohydrates, without affecting the specific volume of the bread. However, higher levels of SSP (4–5%) lead to a notable decrease in the specific volume of burger buns, indicating a threshold beyond which the quality may decline. The incorporation of steamed squid powder (SSP) into burger buns has shown a notable enhancement in sensory properties, with consumer acceptability scores ranging from 8.38 to 8.61. The highest scores were linked to buns containing 3, 4, and 5% SSP, indicating a favorable consumer response.

## Data Availability

The original contributions presented in the study are included in the article/supplementary material, further inquiries can be directed to the corresponding author.
